# A novel wireless implant for central venous pressure measurement: First animal experience

**DOI:** 10.1016/j.cvdhj.2020.10.004

**Published:** 2020-10-29

**Authors:** Tejaswini Manavi, Patricia Vazquez, Helen O’Grady, Jerson Martina, Michael Rose, Douglas Nielsen, David Fitzpatrick, Omid Forouzan, Michael Nagy, Faisal Sharif, Haroon Zafar

**Affiliations:** ∗ Cardiovascular Research & Innovation Centre, National University of Ireland Galway, Galway, Ireland; † Lambe Institute for Translational Research, School of Medicine, National University of Ireland Galway, Galway, Ireland; ‡ Endotronix, Inc, Lisle, Illinois; § Endotronix Ireland Limited, Dublin, Ireland; ‖ BioInnovate Ireland, Galway, Ireland; ¶ Department of Cardiology, University Hospital Galway, Galway, Ireland; # CÚRAM-SFI Centre for Research in Medical Devices, Galway, Ireland

**Keywords:** Central venous pressure, Heart failure, Hemodynamic sensor, Wireless hemodynamic monitoring, Wireless implant

## Abstract

**Background/Objective:**

Central venous pressure (CVP) serves as a surrogate for right atrial pressure, and thus could potentially predict a wider range of heart failure conditions. The purpose of this work is to assess CVP, through an implantable sensor incorporated with a novel anchor design, in the inferior and superior vena cava of an animal model.

**Methods:**

Two animals (Dorset sheep) were implanted with sensors at 3 different locations: inferior vena cava (IVC), superior vena cava (SVC), and pulmonary artery (PA). Two sensors with distinct anchor designs considering anatomical requirements were used. A standard PA sensor (trade name Cordella) was deployed in the PA and SVC, whereas a sensor with a modified cylindrical anchor with various struts was designed to reside in the IVC. Each implant was calibrated against a Millar catheter reference sensor. The ability of the central venous sensors to detect changes in pressure was evaluated by modifying the fluid volume of the animal.

**Results:**

The sensors implanted in both sheep were successful, which provided an opportunity to understand the relationship between PA and CVP via simultaneous readings. The mapping and implantation in the IVC took less than 15 minutes. Multiple readings were taken at each implant location using a hand-held reader device under various conditions. CVP recorded in the IVC (6.49 mm Hg) and SVC (6.14 mm Hg) were nearly the same. PA pressure (13–14 mm Hg) measured was higher than CVP, as expected. The SVC waveforms showed clear beats and respiration. Respiration could be seen in the IVC waveforms, but not all beats were easily distinguishable. Both SVC and IVC readings showed increases in pressure (3.7 and 2.7 mm Hg for SVC and IVC, respectively) after fluid overload was induced via extra saline administration.

**Conclusion:**

In this work, the feasibility of measuring CVP noninvasively was demonstrated. The established ability of wireless PA pressure sensors to enable prevention of decompensation events weeks ahead can now be explored using central venous versions of such sensors.

**Key Findings**

• Central venous pressure (CVP) was assessed in the inferior and superior vena cava of an animal model through an implantable sensor incorporated with a novel anchor design.
• The ability of the central venous sensors to detect changes in pressure was evaluated by modifying the fluid volume of the animal.
• The relationship between pulmonary artery pressure and CVP were studied via simultaneous readings.
• The feasibility of measuring CVP noninvasively was demonstrated.
• The established ability of wireless pulmonary artery pressure sensors to enable prevention of decompensation events weeks ahead can now be explored using central venous versions of such sensors.

## Introduction

Heart failure (HF) is a widespread disease affecting more than 37.7 million people worldwide^1^ and is a main cause of hospitalization in the United States and Europe, with more than 1 million per year in both regions.^2^ Hospitalization of HF patients is frequently followed by readmission and death.^2^, ^3^, ^4^

Most advanced and decompensated HF patients develop congestive HF,^5^ when heart overload leads to accumulation of fluid and pressure around itself and its periphery. Congestion is a direct consequence of cardiac muscle dysfunction, owing to either reduced, preserved, or mid-range HF. In the presence of impaired myocardial pumping, lower perfusion of arterial blood volume activates several neurohormonal mechanisms, which increase heart filling pressures and promote retention of fluids. The presence of congestion exacerbates the progressive deterioration of HF syndrome, significantly shortening life expectancy,^6^^,^^7^ and represents the leading cause of hospitalization in these patients, even ahead of low cardiac output.^4^^,^^5^^,^^8^^,^^9^, ^10^, ^11^

The advent of implantable hemodynamic sensors allows continuous outpatient monitoring of cardiac pressures. Pressure-guided therapy has helped to control and reduce high filling pressures and, as a consequence, congestion.^12^, ^13^, ^14^ In addition, the presence of elevated ventricle filling pressures has been shown to happen days or weeks in advance of an acute HF episode.^8^^,^^15^ Timely therapeutic interventions can often prevent rehospitalization.^12^

Presently, 2 main strategies have prevailed in implantable hemodynamic monitoring. The first one relies on the measurement of pulmonary artery pressure (PAP), which is a surrogate of left atrial pressure (owing to the low resistance in the pulmonary vascular bed), and therefore of left ventricular filling pressure (in the absence of obstructive lesions between left atrium and left ventricle).^16^

The CardioMEMS PAP hemodynamic implant (by Abbott) was the first of its kind to prove the effectiveness of pressure-guided therapy based in PAP values, showing a 39% reduction in rehospitalization.^12^ More recently, the Cordella™ implant (by Endotronix, Inc) has advanced the basic technology.^17^

A second strategy, followed by several implantable hemodynamic sensors, is to measure left atrial pressure (LAP) in situ. This strategy requires intrathoracic implantation, which in the past created implant-related complications that stopped the clinical trial of the first LAP device (HeartPOD, by Abbott).^18^ Nevertheless, results from this trial showed a similar reduction in rehospitalization (41%). New devices are embracing the LAP technology, with V-LAP by Vectorious Medical Technologies^19^ and CardioMon by Integrated Sensing Systems,^20^ both in first-in-human studies.

Despite these advances, all reported hemodynamic monitoring systems tend to focus on the left-side pressures of the heart. The study of HF syndrome traditionally centered on dysfunction in the left side of the heart, with the right side following at a later stage. But more recent work considers the interdependence of both sides. The presence of elevated right heart pressures predicts a very poor outcome in HF owing to a marked increase of mortality.^21^^,^^22^

Central venous pressure (CVP) could represent a valuable hemodynamic parameter to indicate potential dysfunction of the right heart. CVP reflects the value of the right atrial pressure. In addition, as right atrium and ventricle are in fluid communication during end diastole, CVP can provide a good estimate of the diastolic right ventricle pressure.

This study is the first, to our knowledge, to propose a wireless CVP hemodynamic implant for pressure-guided therapy in HF patients. The aim of this work is to assess feasibility of the implantation procedure, anchoring, safety, and applicability of the technology within the venous system.

## Methods

### Implanted system in pulmonary artery and superior veins

For implantation sites in the pulmonary artery (PA) and the superior veins (SV), the acute experiment used the existing Cordella system (Endotronix, Inc, Lisle, IL). This system has been successful in measuring PAP in HF patients and is currently undergoing pivotal clinical trials (ClinicalTrials.gov Identifier: NCT04089059). It consists of a permanently implanted sensor and a handheld external reader unit that the patient uses daily at home to take an 18-second PA pressure reading.

The Cordella sensor is designed for implantation in the downturn of the right PA (or right interlobar PA). Its implantation is minimally invasive and can be done as a part of a right heart catheterization procedure through the femoral or jugular vein. The distal anchor’s length and angle prevent the device from migrating through the PA in any direction. On the other hand, the proximal anchor provides the appropriate outward radial force to keep the sensor stable and apposed directly to the vessel wall.

### Implanted system in inferior vena cava

The inferior vena cava (IVC) is an attractive alternative location for a CVP-measuring implant, featuring simple geometry, sufficient width to reduce risk of vessel occlusion, and direct, minimally invasive surgical access from the femoral vein.

**Figure 1:**
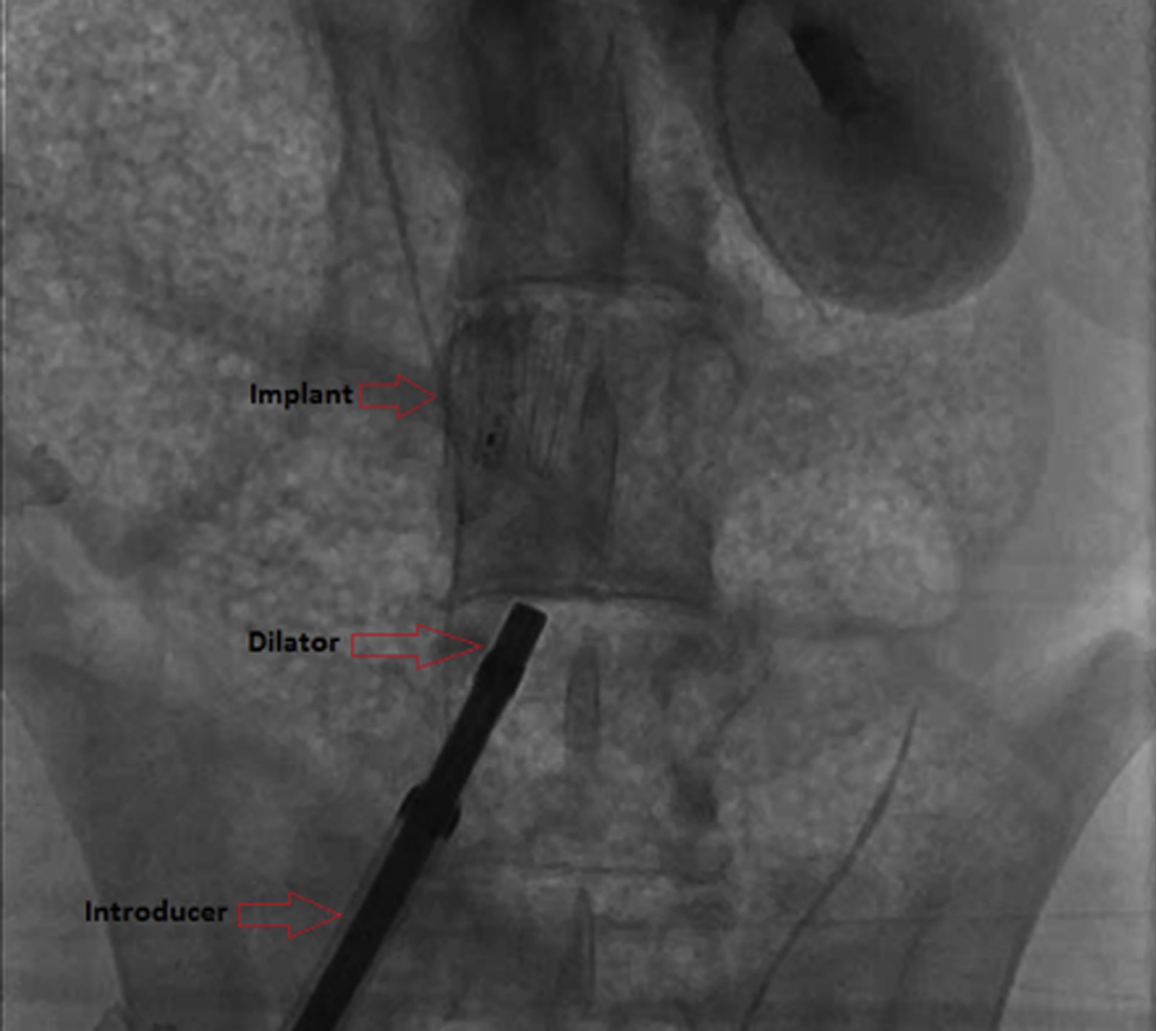
D2 sensor implanted in the D2 area of the inferior vena cava, against a flow of contrast traveling up the vessel.

Analysis of computed tomography (CT) imaging from patients with peripheral vascular diseases led to selection of the section of the IVC between the lower renal vein and the iliac bifurcation (“D2” in Figure 1) as the target implantation site. Its straight geometry is agreeable to an anchor design similar to that of well-established vascular stents or IVC filters, and it avoids risks associated with obstructing the renal veins.

It presents an average diameter of 20 mm (average of 16 CT scans), although from literature the IVC can dilate to as much as 30 mm diameter.^23^^,^^24^ IVC distensibility is an important design consideration and is regarded as an index of the increases in central volume and pressure.^25^

A previous study by Brinkley^26^ compared IVC diameter in patients with and without chronic HF. The primary objective was to determine whether distension of the great veins is a marker on imaging when congestion is the hallmark of HF. The diameter of IVC in patients with chronic HF was in the range of 12–35 mm.^26^ Distensibility numbers were recorded from a study that used methods such as head-down tilt and lower body positive pressure to dilate the IVC. These methods saw a notable ∼15% increase in diameter after distension.^25^ The Brinkley data further inform the operational range of vessel diameters over which an IVC-based sensor’s anchors must function.

Delivery of the D2 system was accomplished by preloading the device into the distal tip of a commercially available 14F introducer. An outer introducer, size 18F, was placed at the femoral access and guided to the D2 target site by angiography. The preloaded 14F introducer was inserted to the deployment site through the outer introducer. A dilator, with its distal tip polished flat, was then used as a push rod to deploy the D2 sensor.

### Implantation protocol

The study conforms to the Guide for the Care and Use of Laboratory Animals and was approved by the Institutional Animal Care and Use Committee. Two healthy adult Dorset sheep were used for this acute study, each weighing approximately 25 kg. The ovine model is preferred for chronic studies of wireless implants for its ability to remain calm and maintain stable position during postimplant readings. This model was selected for the acute study to allow better translation of results to future chronic studies. Each animal was anesthetized and implanted with up to 3 sensors: PA, SV, and IVC. The right PA was accessed from the femoral vein and implanted using a Cordella catheter and implant. The IVC was also accessed from the femoral vein, implanted with a Cordella sensor with modified “D2” anchors and the introducer / pushrod delivery system described above. The superior vein anatomy was accessed from the left external jugular (LEJ) vein, and angiographic mapping identified a suitable “L”-shaped junction between the LEJ and bijugular trunk as the target anatomy. This location was accessed from the jugular vein, using a Cordella delivery catheter to place a standard-anchor implant. In situ wireless readings from each implant were compared with simultaneous pressure readings from a catheter-based Millar (Mikro-Cath™ 825 0101; Millar Instruments, Inc, Houston, TX) reference sensor located near the sensor under test.

## Results

### Implantation in IVC

Angiographic mapping located the lower renal vein entry to the IVC and the iliac bifurcation, and the implant was deployed using the introducer system described above. The sensor deployed in a controlled manner and the anchor conformed to the diameter of the IVC. Figure 1 shows the successfully deployed implant in the “D2” area of the IVC. Anatomical landmark identification and implantation was straightforward, with the latter requiring less than 15 minutes.

### Implantation in the LEJ

**Figure 2:**
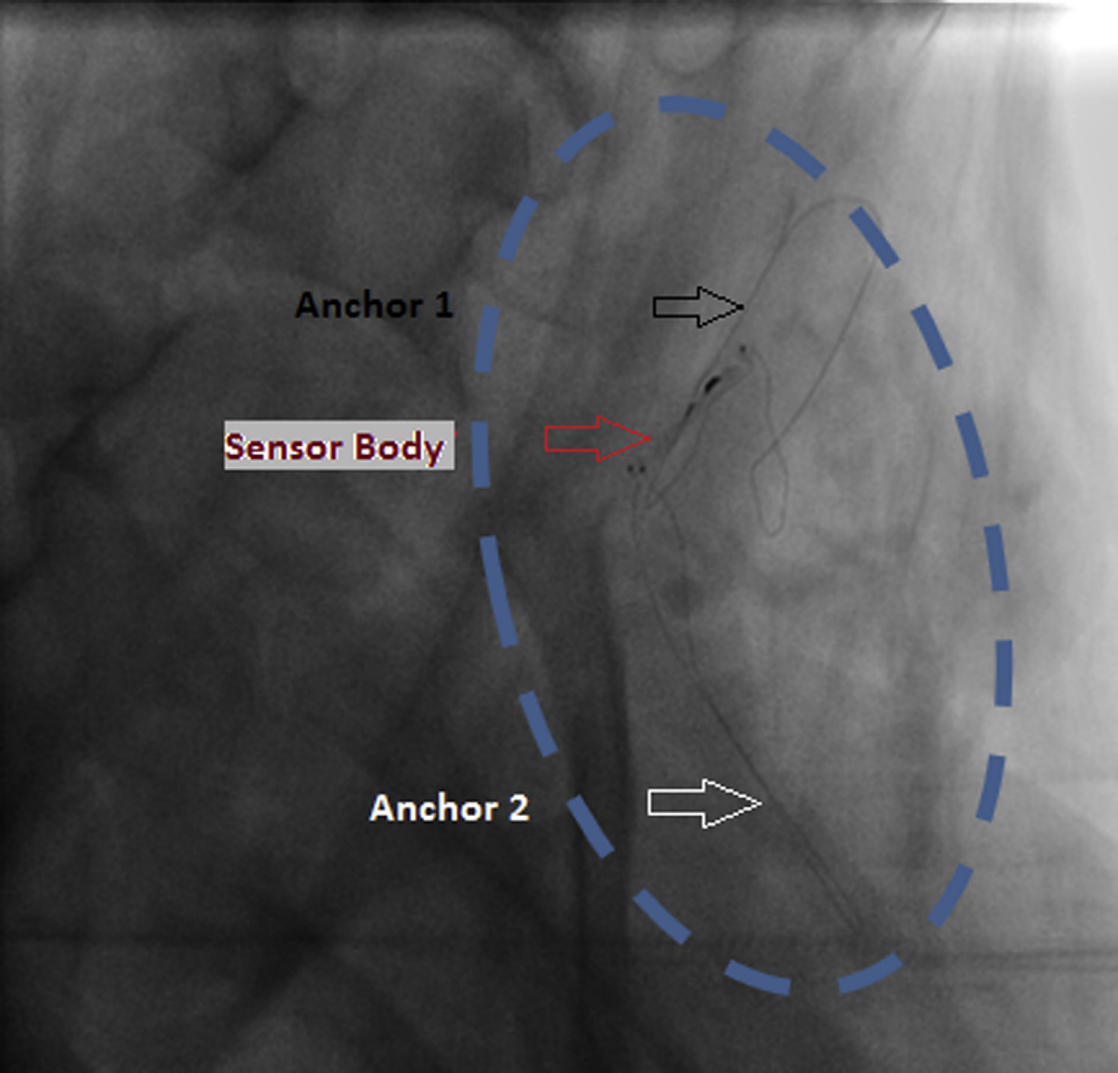
Angiograms of jugular implantation procedure. The implant is placed with distal anchor in the bijugular trunk and proximal anchor in the left exterior jugular vein.

After location of the bijugular trunk junction with contrast and use of quantitative angiography to ensure proper vessel sizing, a guidewire was placed through the LEJ and into the brachial vein. The Cordella delivery system followed the guidewire and deployed the sensor such that its long distal anchor extended into the brachial vein, while its body and proximal anchor remained in the LEJ, as shown in Figure 2. The implantation was straightforward, requiring less than 10 minutes. No modifications to the Cordella delivery catheter or basic procedure were required.

Table 1 provides information on the hemodynamic parameters of the 2 animals recorded during the implantation procedure.

**Table 1:** Information on the hemodynamic parameters of the 2 animals recorded during the implantation procedure

No. of subjects	Heart rate (bpm)	Respiration rate (bpm)	Blood pressure (mm Hg)		Oxygen saturation - SaO2 (%)	Temperature (°F)
			Systolic	Diastolic		
1	109	13	128	87	96	96.6
2	104	7	144	107	99	97

bpm = beats per minute.

### Readings

Multiple individual readings were taken at each implant location under various conditions, using the handheld Reader device. Each implant was calibrated against an in situ catheter-based Millar (Mikro-Cath 825 0101) reference sensor.

**Figure 3:**
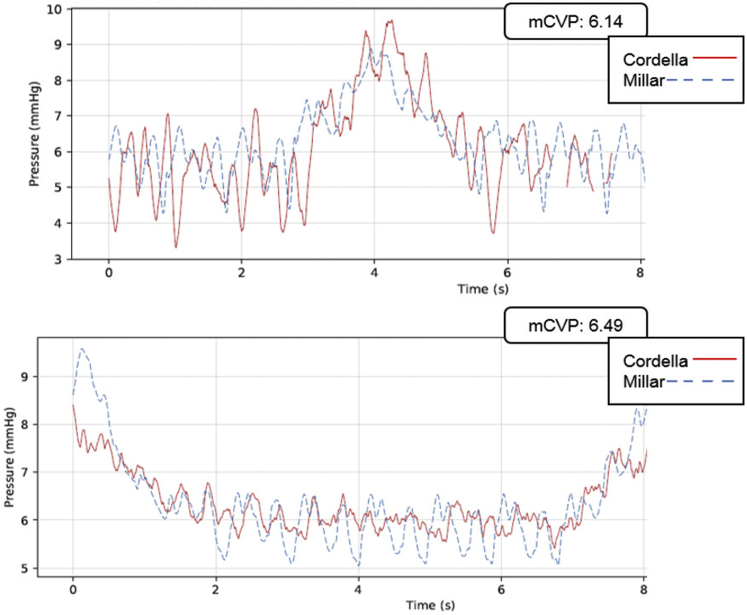
Nonsimultaneous left exterior jugular vein (top) and inferior vena cava (bottom) wireless readings (Cordella [Endotronix, Inc, Lisle, IL], in red) compared to reference catheter readings (Millar [Millar, Inc, Houston, TX], in blue).

Figure 3 provides examples of wireless sensor readings compared to the Millar reference, obtained from the 2 central venous sites, taken at 2 different times. The top plot is from the LEJ (superior vena cava [SVC]) implant and the bottom from the IVC implant.

**Figure 4:**
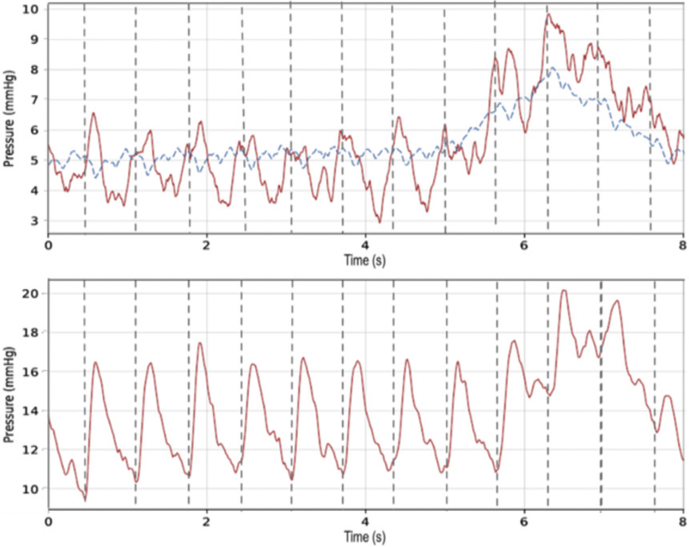
Simultaneous left exterior jugular vein (top) and pulmonary artery (bottom) readings. Red lines are wireless implant readings; the blue line is the Millar (Millar, Inc, Houston, TX) reference.

Figure 4 provides examples of wireless sensor readings compared to the Millar reference, obtained from the 2 central venous sites, taken at 2 different times. The top plot is from the LEJ (SVC) implant and the bottom from the IVC implant.

The SVC plot clearly illustrates the cardiac and respiration cycles. The 2 waveforms are not perfectly time synced owing to small differences in location of the sensors. The reported pressures show good agreement for state-of-the-art in situ pressure sensors.

The IVC waveforms present a smaller amplitude than the LEJ. Both waveforms show similar average pressure. The IVC plot indicates beats and respiration of the 2 measurements (Cordella and Millar). As with the upper plot, sensor performance for the test device and the reference are within expected accuracy limits.

**Figure 5:**
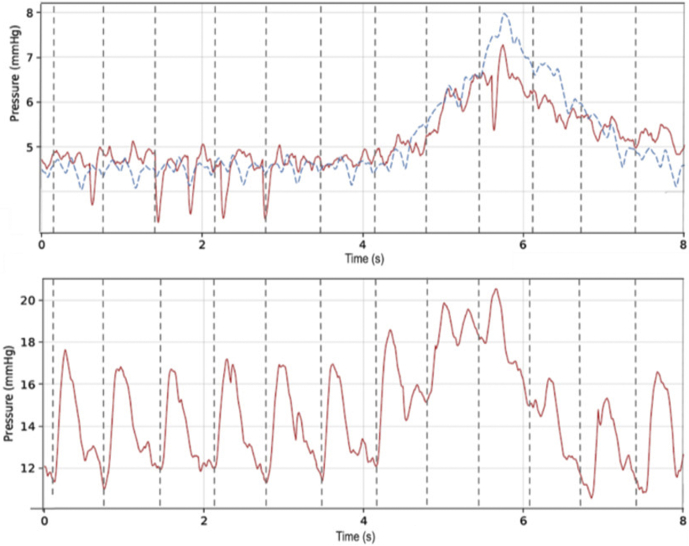
Simultaneous inferior vena cava (above) and pulmonary artery (below) readings. Red lines are wireless implant readings; the blue line is the Millar (Millar, Inc, Houston, TX) reference.

Simultaneous readings were also taken for implants in LEJ vs PA, as shown in Figure 4. The upper LEJ plot shows the wireless sensor output in red and catheter reference measurement in blue. The appearance of peaks in the LEJ waveform may be related to radiofrequency crosstalk between the LEJ and PA sensors, which are in close physical proximity in this animal model. Similarly, the waveforms in Figure 5 were measured simultaneously from the IVC and PA sensors. Table 2 summarizes average pressures during the simultaneous readings.

**Table 2:** Mean pressure values (mm Hg) in pulmonary artery, superior vena cava, and inferior vena cava

	PA	SVC	IVC
Animal 1	---	8.3	8.3
Animal 2	---	6.1	6.5
Simultaneous measurements†	13.6	5.5	---
Simultaneous measurements†	14.5	---	6.1
Extra saline‡	---	12	10.97

IVC = inferior vena cava; PA = pulmonary artery; SVC = superior vena cava.

† Simultaneous measurements were taken in animal 2.

‡ Extra saline fluid was administered to animal 1.

A mild increase in systemic pressure was induced by administering 1 liter of saline fluid intravenously. After about 20 minutes, the mean pressure readings for the SVC and IVC increased from 8.3 mm Hg to 12 and 11 mm Hg, respectively (Table 2).

### Quantitative data analysis

**Figure 6:**
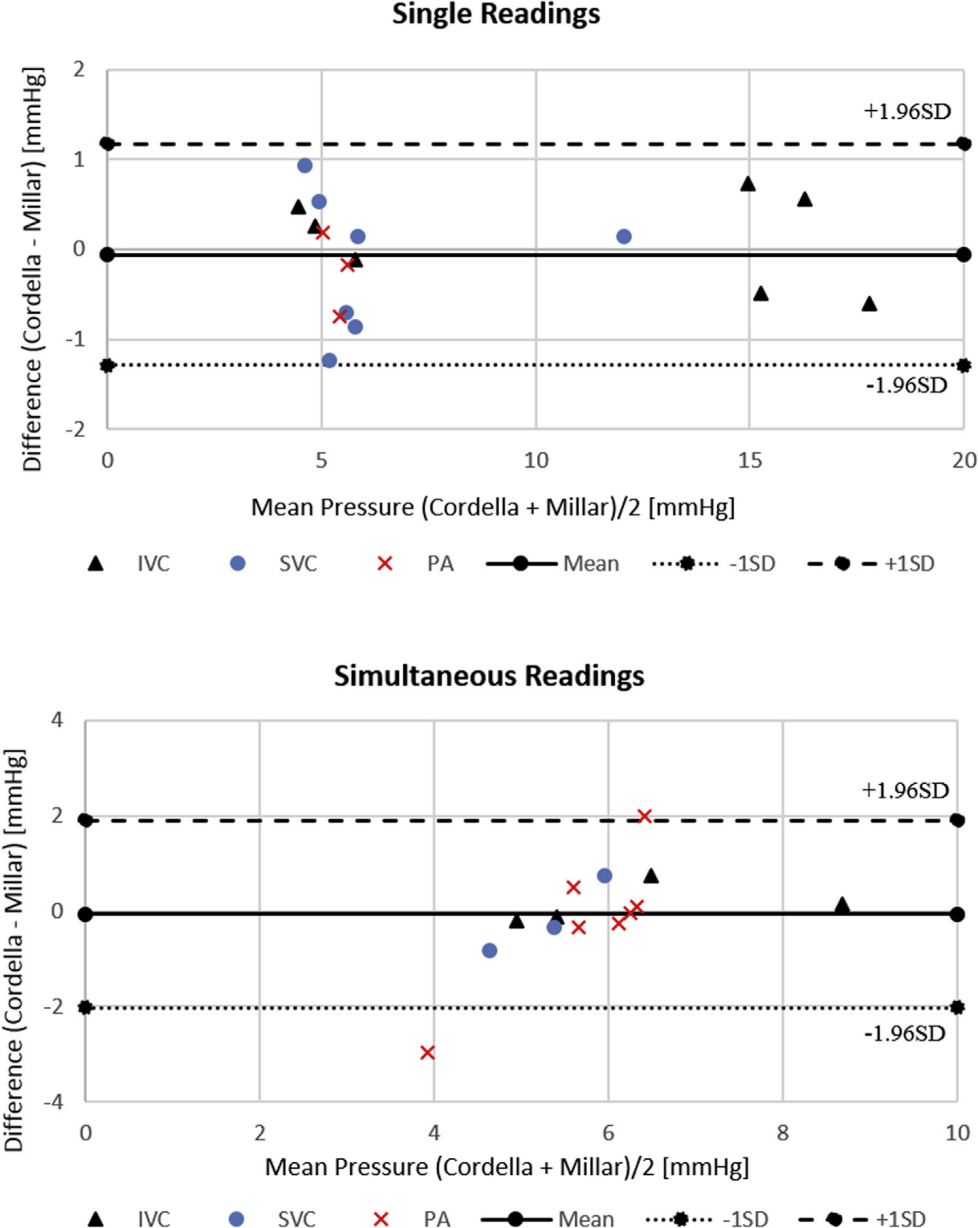
Bland-Altman comparison between Cordella (Endotronix, Inc, Lisle, IL) and reference Millar (Millar, Inc, Houston, TX) sensor; single (*top*) and simultaneous (*bottom*) readings at different locations (inferior vena cava [IVC], superior vena cava [SVC], pulmonary artery [PA]).

This study analyzed 2 sets of data, 1 corresponding to the Cordella sensor measurement and the other to the gold-standard Millar. Both were measured at the same time but at different anatomical locations (IVC, SVC, and PA). In order to more readily observe the differences between the 2 measurements, 2 Bland-Altman plots are introduced to compare the means of each pair of measurements vs the difference between the measurements. One of the plots illustrates single or individual IVC, SVC, and PA readings measured from a single Reader device, whereas the other shows simultaneous readings between the 3 locations measured using 2 Reader devices (1 held at IVC, the other at SVC or PA). These simultaneous measurements were performed to evaluate the accuracy of Cordella when 2 of them were located few centimeters away from each other. Figure 6 shows 2 Bland-Altman plots with single and simultaneous pressure measurements. The simultaneous measurements are broken into a separate accuracy plot, as there is potential for simultaneous reading to generate crosstalk between sensors in such close proximity. This could affect the accuracy of the pressure readings.

Figure 6 indicates that there is good agreement between Cordella and gold-standard Millar measurements. The data points are clustered around the mean of differences and within 2 standard deviations of the mean. Also, the IVC and SVC mean pressures are almost similar. This proves our hypothesis that the CVP in the inferior and superior veins is similar.

### Necropsy

Necropsy inspection of both animals revealed no indication of vessel trauma at the implant sites or along the catheterization pathway for any of the central venous implants. All implants were intact after retrieval, and anchors retained their original deployed shape. No indications of thrombus were observed, consistent with previous experience with this type of implant in the PA.

## Discussion

This study considers the use of a central venous hemodynamic implant for monitoring of heart filling pressures, either as an alternative or a complement to established PAP implants. We have demonstrated successful translation of an existing wireless pressure measurement system from the PA to 2 central venous locations. Implantation was safe, expedient, and straightforward for both locations. The unmodified angular interference anchor, though designed for use in the PA, seated well in the junction of the LEJ and bijugular trunk, and remained in place even when dislodgement was attempted by prodding with a catheter. The IVC implants, featuring the “D2” anchors, remained stable throughout the several hours required for each acute procedure. Probing with a catheter, however, caused them to migrate all the way up to the level of the left kidney. This could have a potential clinical significance and highlights the need to avoid contact of any delivery system with the deployed sensor for future interventions. Future scope may involve efforts in the improvement of IVC anchor design by increasing the outward radial force of anchor wire or incorporating hooks or barbs to prevent migration or dislodgement.

Considerations for superior vs inferior target location include potential obstruction of a catheter pathway or device in future procedures. The external jugular (EJ) location may have an advantage in this respect. EJ access is associated with a low rate of severe mechanical complications compared to femoral access.^27^^,^^28^ Although the EJ/bijugular trunk junction was used opportunistically in this study, it is believed that other acceptable junctions exist in the ovine and human venous systems, requiring little or no modification of the existing angular interference anchors.

The anchors’ “angular interference fit” design concept may be well suited to other angular features in the vasculature, including several steep angles typically present in the superior venous system, for example between junctions of the jugular, brachiocephalic, brachial, or subclavian veins, as well as the SVC. Bench testing using full-scale 3D models created from human CT scans showed promise in several of these locations.

The data collected in the EJ and IVC showed typical right atrial pressure waveforms, with identifiable systolic and diastolic peaks and good agreement with the reference sensors. In addition, simultaneous recordings of the PA and central venous implants were demonstrated. The smaller pulse pressures in the central venous system are at the limit of dynamic resolution of both the wireless and the catheter-based sensors, hence not all waveforms provide clear distinction between actual venous pressure pulses and system noise. Pressure values averaged over several seconds, including readings taken with breathing suspended, showed acceptable accuracy.

The animals used in this study were healthy Dorset sheep selected to provide a baseline CVP measurement, as a reference for future studies that may involve animals with induced pulmonary or systemic congestion. One of the objectives was also to confirm that the pressure values in the IVC and EJ locations are similar. Results shown in Table 2 indicated a difference of 1.03 mm Hg.

### Limitations

As a first-of-its-kind feasibility study, this exercise was limited to a small sample size. The sample was appropriate to gain first indications of the device’s ability to deploy safely and measure blood pressure wirelessly from within the central venous system. As detailed literature on the ovine central venous system is limited, the study also identified optimum locations for device implantation. The selected sample size was adequate for these qualitative objectives. Larger, statistically significant populations will be employed in later stages of this research.

The animal experiment is supported by results and analysis of human data, as the ultimate goal is implantation in humans through translational research. Various locations of human IVC and SVC were selected using CT scans, and bench experiments on a silicone vasculature derived from a human CT scan were performed to determine an optimal location for implantation in vivo. These efforts led to selection of ovine deployment sites that corresponded anatomically to attractive sites in human vasculature.

This study also limits hemodynamic characterization and identification of typical patterns in the waveform (a, c, x, v, y), which will be addressed in later stages of this research. The current Cordella pressure data is filtered by a 5 Hz second-order low-pass filter. This limits the characterization of higher-frequency IVC or SVC waveform properties (typically around 20 Hz) by the Cordella pressure signal. Nevertheless, the aim of the pressure measurement was to assess mean IVC and SVC pressure rather than the hemodynamic characteristics of such a waveform. The mean pressures may facilitate the assessment of clinically relevant changes associated with hemodynamic congestion.^29^

## Conclusion

Pressure guided therapy based on hemodynamic implantable sensors has shown effectiveness in reducing and controlling congestive HF. We demonstrated excellent translation of an existing implantable wireless pressure sensing system from the PA to 2 locations in the central venous system. This system will provide researchers with a new tool to explore clinical applications of safe, simple, on-demand acquisition of this important hemodynamic parameter.

### Funding Sources

The work leading to this publication has received funding from 10.13039/501100001588Enterprise Ireland and the European Union’s Regional Development Fund (Innovation Partnership Programme, Grant Number: IP20180755). The opinions, findings, and conclusions or recommendations expressed in this material are those of the authors and neither Enterprise Ireland nor the European Union are liable for any use that may be made of information contained herein. Dr Haroon Zafar is supported by 10.13039/501100001602Science Foundation Ireland “Technology Innovation Development Award” (Grant Number: 18/TIDA/6017). Tejaswini Manavi is supported by an Irish 10.13039/501100002081Research Council “Enterprise Partnership Scheme Postgraduate Scholarship” (Grant Number: EPSPG/2020/310). Disclosures: J. Martina, M. Rose, D. Nielsen, O. Forouzan and M. Nagy work for Endotronix, Inc. D. Fitzpatrick works for Endotronix Ireland Limited. There is no conflict of interest for T. Manavi, P. Vazquez, H. O’Grady, F. Sharif, and H. Zafar.
